# Supplementary material for: New perspectives on ‘Breathomics’: metabolomic profiling of non-volatile organic compounds in exhaled breath using DI-FT-ICR-MS
Source: Commun Biol. 2024 Mar 2;7:258. doi: 10.1038/s42003-024-05943-x (PMC10908792; doi:10.1038/s42003-024-05943-x)
Supplement: Supplementary file 2 — Supplementary Information [file 42003_2024_5943_MOESM2_ESM.pdf]

Supplementary Information

New Perspectives on 'Breathomics': Metabolomic Profiling of Non-Volatile Organic Compounds in Exhaled Breath using DI-FT-ICR-MS

Madiha Malik[1,*], Tobias Demetrowitsch[2,3,*], Karin Schwarz[2,3] and Thomas Kunze[1]

[1] Department of Clinical Pharmacy, Institute of Pharmacy, Kiel University, Kiel, Germany.

[2] Institute of Human Nutrition and Food Science, Food Technology, Kiel University, Kiel, Germany.

[*] contributed equally

Corresponding Authors:

Madiha Malik (mmalik@pharmazie.uni-kiel.de), **Thomas Kunze** (tkunze@pharmazie.uni-kiel.de)

1. Supplementary description of the exhaled breath collection device (Supplementary Figure 1)

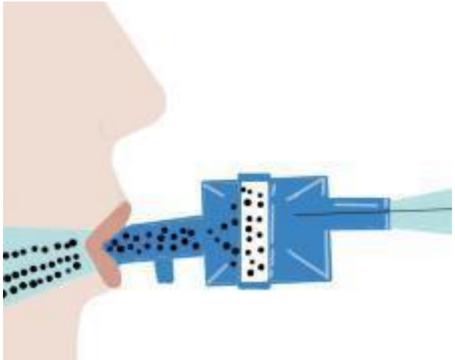

The exhaled breath samples were collected using a simple, ready-to-use device (SensAbues®, Stockholm, Sweden, see: http://sensabues.com/product) consisting of a mouthpiece and a polymeric electret filter enclosed in a plastic collection chamber. As depicted, the patient exhales through the mouthpiece of the device. The filter inside the device enables the collection of exhaled breath. After sampling, the device is sealed at both ends and samples are stored at -80 °C. The electret air filter of the device is used for further multistep extraction of metabolites.

2. Supplementary Table 1

Supplementary Table 1: The ten most relevant (most significant) metabolites in all three statistical tests (PLS-DA, Volcano plot, Random forest) that were identified by means of VENN diagram analysis. This table characterizes metabolites by VIP scores, adjusted p-values and mean decrease accuracy values.

		**PLS-DA**	**Volcano plot**	**Random forest**
**Putative identity**	**Chemical formula**	**VIP score**	**adjusted p-value**	**Mean decrease accuracy**
Phthalic anhydride	$C_8H_4O_3$	3.3428	6.04E-07	0.0010161
1-Deoxy-D-glucitol	$C_6H_{14}O_5$	3.0204	1.05E-06	0.0058354
2-butoxyethanol	$C_6H_{14}O_2$	1.9874	2.54E-08	0.0053111
Xestoaminol C	$C_{14}H_{31}NO$	2.6568	5.92E-10	0.00152
1-Octanesulfonic acid	$C_8H_{18}O_3S$	1.9726	5.15E-12	0.010006
Koenoline	$C_{14}H_{13}NO_2$	3.6168	6.96E-10	0.0012252
7-[3-(3-Hydroxyoct-1-en-1-yl)-6-thiabicyclo[3.1.1]heptan-2-yl]hept-5-enoate	$C_{21}H_{34}O_3S$	2.6755	9.77E-07	0.00078723
2.8-Dibenzylcyclooctanone	$C_{22}H_{26}O$	4.3317	1.23E-08	0.0007889
Desethylchloroquine	$C_{16}H_{22}ClN_3$	3.2392	6.42E-08	0.0024541
azepan-2-one	$C_6H_{11}NO$	2.2007	1.77E-21	0.015092
Cyclododecanol	$C_{12}H_{24}O$	1.9448	5.15E-12	0.0062865
