# Supplementary material for: New perspectives on ‘Breathomics’: metabolomic profiling of non-volatile organic compounds in exhaled breath using DI-FT-ICR-MS
Source: Commun Biol. 2024 Mar 2;7:258. doi: 10.1038/s42003-024-05943-x (PMC10908792; doi:10.1038/s42003-024-05943-x)
Supplement: Supplementary file 3 — Description of Additional Supplementary Files [file 42003_2024_5943_MOESM3_ESM.pdf]

Description of Additional Supplementary Files

File name: Supplementary Data

Description: The raw dataset, including the population characteristics and the source data behind the graphs in the paper.
