# Supplementary material for: New perspectives on ‘Breathomics’: metabolomic profiling of non-volatile organic compounds in exhaled breath using DI-FT-ICR-MS
Source: Commun Biol. 2024 Mar 2;7:258. doi: 10.1038/s42003-024-05943-x (PMC10908792; doi:10.1038/s42003-024-05943-x)
Supplement: Supplementary file 5 — Reporting Summary [file 42003_2024_5943_MOESM5_ESM.pdf]

nature portfolio

Corresponding author(s): Madiha Malik, Thomas Kunze

Last updated by author(s): Jan 29, 2024

Reporting Summary

Nature Portfolio wishes to improve the reproducibility of the work that we publish. This form provides structure for consistency and transparency in reporting. For further information on Nature Portfolio policies, see our Editorial Policies and the Editorial Policy Checklist.

Statistics

For all statistical analyses, confirm that the following items are present in the figure legend, table legend, main text, or Methods section.

n/a	Confirmed	
☐	☒	The exact sample size (*n*) for each experimental group/condition, given as a discrete number and unit of measurement
☐	☒	A statement on whether measurements were taken from distinct samples or whether the same sample was measured repeatedly
☐	☒	The statistical test(s) used AND whether they are one- or two-sided<br>*Only common tests should be described solely by name; describe more complex techniques in the Methods section.*
☐	☒	A description of all covariates tested
☐	☒	A description of any assumptions or corrections, such as tests of normality and adjustment for multiple comparisons
☐	☒	A full description of the statistical parameters including central tendency (e.g. means) or other basic estimates (e.g. regression coefficient) AND variation (e.g. standard deviation) or associated estimates of uncertainty (e.g. confidence intervals)
☐	☒	For null hypothesis testing, the test statistic (e.g. *F*, *t*, *r*) with confidence intervals, effect sizes, degrees of freedom and *P* value noted<br>*Give P values as exact values whenever suitable.*
☒	☐	For Bayesian analysis, information on the choice of priors and Markov chain Monte Carlo settings
☒	☐	For hierarchical and complex designs, identification of the appropriate level for tests and full reporting of outcomes
☒	☐	Estimates of effect sizes (e.g. Cohen's *d*, Pearson's *r*), indicating how they were calculated

Our web collection on statistics for biologists contains articles on many of the points above.

Software and code

Policy information about availability of computer code

Data collection	Microsoft Excel 2016, R 4.3
Data analysis	Origin 2020, R 4.3, MetaboScape 2021b, MetaboAnalyst 5.0

For manuscripts utilizing custom algorithms or software that are central to the research but not yet described in published literature, software must be made available to editors and reviewers. We strongly encourage code deposition in a community repository (e.g. GitHub). See the Nature Portfolio guidelines for submitting code & software for further information.

Data

Policy information about availability of data

All manuscripts must include a data availability statement. This statement should provide the following information, where applicable:

- Accession codes, unique identifiers, or web links for publicly available datasets
- A description of any restrictions on data availability
- For clinical datasets or third party data, please ensure that the statement adheres to our policy

The (pre-processed) raw dataset and population characteristics are available in the Supplementary Data. Statistical analyses of the dataset can be performed using MetaboAnalayst. All other data are available from the corresponding authors on reasonable request. Further inquiries can be directed to mmalik@pharmazie.uni-kiel.de or tkunze@pharmazie.uni-kiel.de.

April 2023

Research involving human participants, their data, or biological material

Policy information about studies with human participants or human data. See also policy information about sex, gender (identity/presentation), and sexual orientation and race, ethnicity and racism.

Reporting on sex and gender	The findings of this study do not apply to only one sex. Herein, the terms „sex“ and „gender“ were used synonymously. The study population was grouped in males (n=51) and females (n=50). Sex and gender was determined based on the biological attribute, matching the self-defined gender. The sex and gender was also considered as part of our analyses, revealing gender/sex-specific metabolic patterns in exhaled breath.
Reporting on race, ethnicity, or other socially relevant groupings	The terms "race, ethnicity or other socially relevant groupings" were not used in the manuscript. This study did not group the study population by race or ethnicity.
Population characteristics	Age: 20-40, normal BMI: 19.0 25.0 kg/m^2, male: n= 51, female: n= 50, healthy volunteers = (1) not diagnosed with chronic conditions or diseases, such as diseases of the airways of the lungs, disorders of the endocrine system, metabolic disorders, as well as those (2) not suffering from an acute (contagious) disease, e.g. infection of respiratory tract like COVID 19. In addition, regular medication (except from oral contraceptives) and acute medication such as antibiotics were exclusion criteria. Moreover, patients with mental disorders and pregnant women were excluded from the study.
Recruitment	Healthy volunteers between 20 and 40 and a normal BMI were recruited. This is a cross-sectional study that included volunteers who responded to an announcement for this study. The announcement was available online, and also placed at different locations at university, different institutes etc. The recruitment of volunteers occured between August 11th 2022 and January 19th 2023.
Ethics oversight	Approved by: The Ethics Committee of the Faculty of Medicine, Kiel University, Germany, (D511/22). Informed consent for scientific use and publication of anonymized data was provided by each volunteer.

Note that full information on the approval of the study protocol must also be provided in the manuscript.

Field-specific reporting

Please select the one below that is the best fit for your research. If you are not sure, read the appropriate sections before making your selection.

☒ Life sciences ☐ Behavioural & social sciences ☐ Ecological, evolutionary & environmental sciences

For a reference copy of the document with all sections, see nature.com/documents/nr-reporting-summary-flat.pdf

Life sciences study design

All studies must disclose on these points even when the disclosure is negative.

Sample size	No sample-size calculation was performed before. This study included 101 healthy volunteers providing 3 replicates. A sample size of 303 was sufficient for this proof of concept study. Male and female participants were recruited and included in the study evenly (female n= 50, male n=51).
Data exclusions	No data was excluded from analyses.
Replication	Three replicates were taken from each participant, all of them were tested and analysed in one laboratory. All samples were prepared identically by one person, analysis was performed in batches (without interruptions). The analysis included quality controls throughout analysis. These quality controls were pooled samples consisting of all samples. They were measured on each analysis day to monitor data quality.
Randomization	Allocation was not random. Since the study only included healthy volunteers, randomization was not applicable. The study population was neither assigned to a treatment nor a control group. The purpose of this proof-of-concept study was to characterize the metabolome in breath of healthy persons.
Blinding	As described above, blinding was not relevant for this study. Only healthy participants were included. The purpose of this proof-of-concept study was to characterize the metabolome in breath of healthy persons.

Reporting for specific materials, systems and methods

We require information from authors about some types of materials, experimental systems and methods used in many studies. Here, indicate whether each material, system or method listed is relevant to your study. If you are not sure if a list item applies to your research, read the appropriate section before selecting a response.

Materials & experimental systems

n/a	Involved in the study
☒	☐ Antibodies
☒	☐ Eukaryotic cell lines
☒	☐ Palaeontology and archaeology
☒	☐ Animals and other organisms
☐	☒ Clinical data
☒	☐ Dual use research of concern
☒	☐ Plants

Methods

n/a	Involved in the study
☒	☐ ChIP-seq
☒	☐ Flow cytometry
☒	☐ MRI-based neuroimaging

Clinical data

Policy information about clinical studies

All manuscripts should comply with the ICMJE guidelines for publication of clinical research and a completed CONSORT checklist must be included with all submissions.

Clinical trial registration

This proof-of-concept study is not an interventional, clinical study investigating clinical outcomes. Therefore, since the study population only consists of healthy controls and no intervention took place, this study was not registered as a clinical trial.

Study protocol

Since only healthy volunteers participated in this study, there is no trial protocol available for this study. The manuscript, however, incorporates inclusion and exclusion criteria and details on recruitment and sample collection.

Data collection

Data was collected by Madiha Malik (corresponding author). Only Madiha Malik has full access to all data provided by participants. Data was collected between August 2022 and January 2023.

Outcomes

No explicit outcome measures. The goals of the study were achieved. This metabolomics study successfully defined the non-volatile core metabolome of exhaled breath in healthy participants, laying the foundation for evaluating clincal biomarkers in breath research.

Plants

Seed stocks

Report on the source of all seed stocks or other plant material used. If applicable, state the seed stock centre and catalogue number. If plant specimens were collected from the field, describe the collection location, date and sampling procedures.

Novel plant genotypes

Describe the methods by which all novel plant genotypes were produced. This includes those generated by transgenic approaches, gene editing, chemical/radiation-based mutagenesis and hybridization. For transgenic lines, describe the transformation method, the number of independent lines analyzed and the generation upon which experiments were performed. For gene-edited lines, describe the editor used, the endogenous sequence targeted for editing, the targeting guide RNA sequence (if applicable) and how the editor was applied.

Authentication

Describe any authentication procedures for each seed stock used or novel genotype generated. Describe any experiments used to assess the effect of a mutation and, where applicable, how potential secondary effects (e.g. second site T-DNA insertions, mosiacism, off-target gene editing) were examined.
